# NARRATIVE EXPLORATIONS OF CARE: THE TRANSFORMATIVE POWER OF CAREGIVING IN THE CONTEXT OF MENTAL HEALTH AND CANCER

**DOI:** 10.1093/geroni/igad104.3484

**Published:** 2023-12-21

**Authors:** Charlotte Weiss, Rachel Johnson-Koenke, Karen Sousa, Connie Ulrich, Karen Hirschman

**Affiliations:** University of Pennsylvania, Philadelphia, Pennsylvania, United States; University of Colorado- Anschutz Medical Campus, Aurora, Colorado, United States; University of Colorado- Anschutz Medical Campus, Aurora, Colorado, United States; University of Pennsylvania, Philadelphia, Pennsylvania, United States; University of Pennsylvania, Philadelphia, Pennsylvania, United States

## Abstract

Embracing the “narrative turn,” we used the post-modern and feminist informed research methodologies of narrative inquiry alongside photo elicitation to co-create an in-depth cancer family caregiver narrative with a 50-year-old daughter who cared for her mother (age 80) affected by lifelong schizophrenia and recent metastatic breast cancer. The participant was invited to share personal photos to support her in co-creating a caregiver narrative within the narrative interview environment. While these photos were not analyzed for content, they prompted the participant to share stories about her private caring experiences and make meaning of them. The I-statements of these stories were then used to create a research poem which explores a life lived alongside a parent with mental health issues, while highlighting the transformation and healing potential that life-limiting cancer introduced to their complex relationship affected by mental health barriers. The I-Poem rendering was analyzed for cognitive and emotional meaning resonant of the human experience. Emergent thematic threads of personal humanistic values include altruism, love, and forgiveness. Additionally, this I-Poem analysis contributes to new knowledge situated in human story telling while capturing a transformational personal experience of caring as an exemplar which resists discrimination and disunity among older persons and their family caregivers on the basis of mental health with concomitant serious illness.

